# Natural Rigid and Hard Plastic Fabricated from Elastomeric Degradation of Natural Rubber Composite with Ultra-High Magnesium Carbonate Content

**DOI:** 10.3390/polym15143078

**Published:** 2023-07-18

**Authors:** Abedeen Dasaesamoh, Kittikhun Khotmungkhun, Kittitat Subannajui

**Affiliations:** Material Science and Engineering Program, School of Materials Science and Innovation, Faculty of Science, Mahidol University, Bangkok 10400, Thailandkittikhun.kho@alumni.mahidol.ac.th (K.K.)

**Keywords:** natural rubber, high filler loading, composite materials, mechanical properties

## Abstract

It is known that natural rubber is an elastomeric polymer; hence, the main uses are usually limited to soft applications. For the process to reverse the elastomeric effect of natural rubber to obtain rigid plastic from a natural material, an ultra-high amount of magnesium carbonate particles was added to the natural rubber to study the effect of magnesium carbonate in the reduction of elastomeric properties. High magnesium carbonate ratios of 80–180 phr were mixed in the natural rubber in the latex form to maximize the mixing capability since it was more difficult to achieve these mixture ratios with only two roll mill or extruder processes. The more magnesium carbonate powders in the composite, the higher torques were measured from the moving die rheometer (MDR) test. The powder was thoroughly mixed inside the composite, which was observed from energy-dispersive X-ray spectrometer (EDX) mapping; however, the matrix of composites was filled with porosity due to the CO_2_ formation when latex with magnesium carbonate was assimilated with acid during the vulcanization process. The strength of the composite dropped, and the elongations were shortened. On the other hand, the hardness of composites was drastically increased. The composite lost the elastomeric property, and the hard natural rubber composites were obtained.

## 1. Introduction

With the deterioration of the environment due to the pollution that humans produce, researchers around the world have begun to realize the methods for rehabilitation. The reduction of synthesized materials, such as polymers, which are manufactured from petrochemical processes, could influence world restoration. Since most rigid plastics in the industries were produced from synthetic polymers, the original monomers were manufactured from chemical synthesis. In order to reduce the impact on the environment due to the harvesting and production processes, additional information on how to obtain natural rigid plastic, which is more environmentally friendly, could be meaningful. Composite materials from natural rubber have been given significant attention by researchers due to their versatile applications, especially in modern green design [1]. Unlike synthetic rubber, which originated from petrochemical processes, natural rubber could be considered a green material and fabricated with overall lower energy consumption. It also provides exceptional flexibility, which could hardly be replaced by an artificial polymer. Hard and rigid materials tend to rely on petroleum-based polymers. In order to explore the extension of natural rubber applications to the hard and non-flexible aspect, elastomeric degradation of natural rubber must be intentionally achieved. With the recent advance of composite technology, the rigidity and hardness of polymer composite could be controlled. The hardness control yields a possibility for natural rubber to obtain high rigidity and hardness for various applications. The hardness of the composite could be determined by the amount of inorganic additive in the polymer [2,3]. To achieve high rigidity and hardness, very high loading of inorganic particles has to be able to blend homogeneously inside the polymer, which was proved by many types of polymer composites. When higher amounts of inorganic additives were loaded, harder composites were obtained, for example, wasted boron in resin and calcium carbonate in polypropylene [4,5]. Moreover, various applications were implemented with this high loading of inorganic additives in polymers, such as refractory and sensors [6,7,8]. Although it was widely known that additives could improve the mechanical property of rubber, most of the additives that were used in commercialized products and research were carbon-based fillers such as carbon black and fly ash [9,10]. The hardness variation and improvement of natural rubber to the point where it is very rigid by using a very high amount of additive is still not thoroughly observed. Since the resource to produce natural rubber is the water-based emulsion known as latex from the tree, the inorganic additive could be added and mixed in the liquid form. Among various kinds of inorganic particles, MgCO_3_ is an additive compound that can be easily found in mining activity and is normally used in refractory applications [11,12,13]. MgCO_3_ was found to assist in the reduction of silane usage for natural rubber, which required silica dispersion [14]. It could also directly influence the mechanical properties of natural rubber [15]. It is interesting to note that research on magnesium carbonate/natural rubber composites had a strong start in the past, but there seems to have been a decline in studies dedicated to this topic in recent years. This shift in research attention is worth considering and further exploring. In this work, various compositions of MgCO_3_ were loaded into the latex to increase the hardness and rigidity of the natural rubber composite.

## 2. Materials and Methods

The natural rubber used in this study was natural rubber latex. The dry rubber content (DRC) was 60%. The vulcanization agents were in dispersion form, including ZnO, Wingstay L, ZDEC, DPG and Sulfur. The filler was MgCO_3_, which had a particle size (D_90_) of 22.6 μm. The natural rubber latex, vulcanization agents, and MgCO_3_ were purchased from Chemical & Materials Co., Ltd., Bangkok, Thailand.

**Figure 1:**
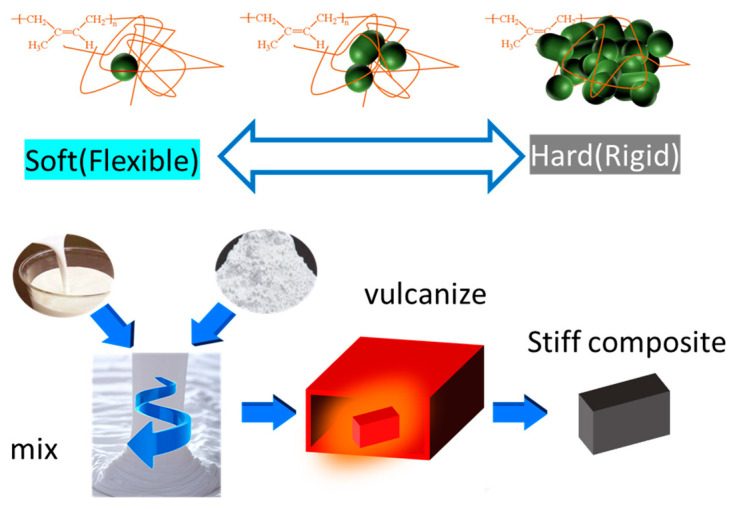
The concept for high MgCO_3_/NR composite preparation.

The MgCO_3_/NR composites were prepared by solution mixing method, as shown in Figure 1. Firstly, the suspensions of MgCO_3_ particles were prepared with DI water and stirred for 1 h. After the homogeneous suspension was obtained, the natural rubber latex, which contained the vulcanized agents, was added into the MgCO_3_ solution and continued stirred for another 5 min. The amount of vulcanization such as ZnO, Wingstay L, ZDEC, DPG and Sulfur were 0.5, 1, 1.5, and 2.5 phr, respectively. The mixture was transferred to a big bowl before coagulation by a 3% acetic acid solution to enhance the speed of polymeric agglomeration. The composite compound was washed to remove acetic acid. To remove water and avoid porosity, the compound was pressed by a compress machine. The samples were dried in an oven. The composites were formed in a square mold under a compress machine at 130 °C. The MgCO_3_ particles were added with various compositions at 80, 100, 120, 140, 160, and 180 phr.

The curing characteristic of the composite was conducted by MDR test at 130 °C. Curing temperature, maximum torque (M_H_), and minimum torque (M_L_) were measured from MDR curves. The compound was cured following curing time (t_c_90). The cross-section morphology of the MgCO_3_/NR composite was observed from SEM images. The MgCO_3_ particle distribution in a natural rubber matrix was examined by the EDX technique. The fire resistance was conducted in horizontal mode. The test samples were cut in 12.7 cm and marked at 2.54 cm and 10.16 cm for a starting point and stopping point, respectively. The specimen was held at one end at a horizontal angle. The flame was ignited to the free end for 30 s or until the flame front reached the 2.54 cm mark. The time that the flame started from the 2.54 cm mark to the second mark at 10.16 cm was recorded. The experiments were measured in three samples for each condition.

For the abrasion test, the test sample diameter was 100 mm. The standard thickness was 6.35–4.00 mm. The test sample was placed on the flat surface of the machine, and two genuine taber abrasive wheels were pressed on the samples with a load of 1000 g on the abrader wheel, which was spinning at 60 rpm for 1000 cycles. The weight losses were measured after the test. The chemical resistance of difference MgCO_3_ loading was evaluated by following ISO10545. This test method was usually used in the chemical resistance test of ceramic tiles at room temperature. The sample was first prepared by cutting into 5 × 5 cm^2^ before being placed in an oven at 80 °C for 3 h. to obtain a constant weight. The chemical resistance of MgCO_3_/NR composite to acidic and alkali solutions was studied. Two different concentrations of KOH and HCL were used. The KOH solution of 30 g/L and 100 g/L were prepared for low- and high-concentration solutions. Additionally, the HCl solution of 3% and 18% concentration were prepared as low- and high-concentration solutions. The samples were placed in a plastic box containing a testing chemical solution as prepared above and kept for 12 days. After 12 days, the samples were taken off the solution, and the surface was cleaned with DI water before testing. The mechanical properties of the obtained composite were characterized by the tensile test and hardness test. Dumb-bell-shaped samples were prepared according to ASTM D 412 for tensile testing. Tensile tests were measured at a cross-head speed of 500 mm/min at room temperature by a universal testing machine (INTRON336). The hardness values were measured with a shore A durometer. The tests were measured for 5 of 5 × 5 cm^2^ samples, and the results were averaged.

## 3. Results

In order to increase the rigidity of natural rubber, a large amount of MgCO_3_ inorganic particles was loaded into the natural rubber. MgCO_3_ in this experiment usually had a Mohs hardness of around 4, which was rather soft compared to other kinds of ceramics such as alumina or silica; however, MgCO_3_ was inorganic material with a higher polarity, which was compatible with the water-based process in this experiment where latex and inorganic particle were blended in liquid form as shown in Figure 1. The inorganic particles could be easily stirred and mixed inside the latex solution. When compared to the addition of a very large amount of inorganic particles in natural rubber by using two roll mills or screw extruder machine, the high-loading MgCO_3_ composite was too stiff, and coagulation occurred inside the machine, which caused damage to the roller and screw. Hence, blending a large amount of inorganic particles in liquid latex rather than mixing them with two roll mills yielded a more favorable process.

**Figure 2:**
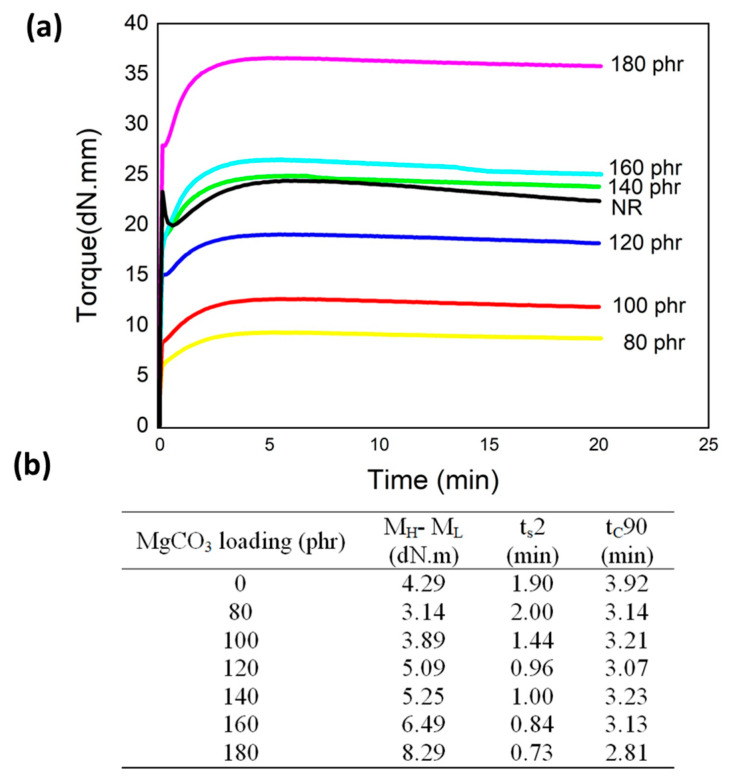
(**a**) Cure curve and (**b**) Vulcanization characteristics of MgCO_3_/NR composites.

The quality of MgCO_3_/natural rubber composite depended mainly on the interaction of particles and the polyisoprene chain, which was related to the crosslink of the polymeric chain around the particles. The vulcanization characterizations were observed from MDR results, as shown in Figure 2a, and vulcanization parameters, including t_s_2, t_c_90, and ∆M, were also listed in Figure 2b. Pure natural rubber provides higher torque than natural rubber with 80 phr, which indicates the degradation of crosslink property due to a high addition of MgCO_3_. When the amount of MgCO_3_ increased, the torque also increased. The crosslink-enhanced influence of MgCO_3_ on the crosslink was expected with a higher maximum torque when the interaction particle and polymer chain were increased at high contents (80–180 phr). The scorch time t_s_2 of composites was shortened when MgCO_3_ was increased, and the cure time t_c_90 was longer with an additional amount of MgCO_3_ particle. This trend from the results implied that by adding more MgCO_3_, the composite started the crosslinking process faster, but the duration when the composite was completely cured was much longer [15,16]. The additional amount of MgCO_3_ offered an advantage for the flow control of the composite to be completely cured inside the mold with less problem about the coagulation before filling the mold due to the fast crosslink. The torque difference (M_H_-M_L_) had also risen up when the MgCO_3_ content increased. The torque was increased as expected due to the particle and polymer chain interactions, which were already mentioned in the former section. Moreover, the addition of MgCO_3_ exhibits a plateau relationship after the torque reaches the maximum value [17]. This behavior indicated that the crosslinking structure was so stable that it was able to withstand the degradation effect at the elevated temperature over curing. In general, adding a very high loading content of MgCO_3_ did not show any sign of adverse effect when observed by the MDR test.

**Figure 3:**
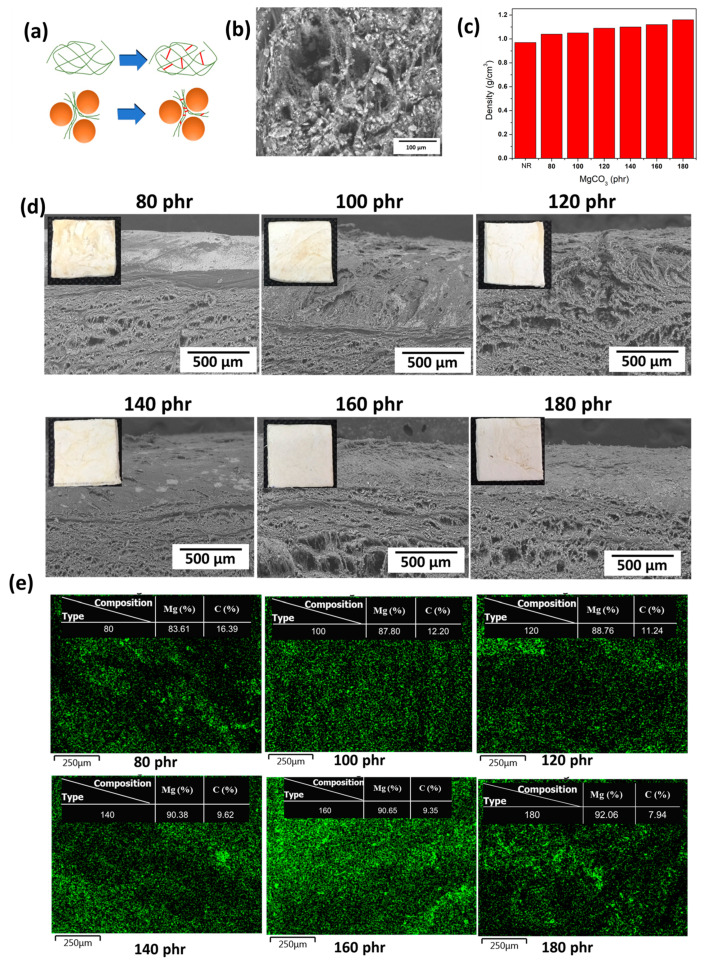
(**a**) Schematic of molecular crosslink in rubber and high-loaded composite; (**b**) SEM image of MgCO_3_ morphology; (**c**) density of the composite with different MgCO_3_ loading; (**d**) SEM image of the composite with different MgCO_3_ loading; and (**e**) EDX of Mg distribution in the composite samples.

According to the results of GPC-SEC presented in Table S1, the molecular weight of MgCO_3_/NR composites, only 0 phr could be diluted in THF (Tetrahydrofuran) after 1 month. This result implied that the high-loaded MgCO_3_ tended to obstruct the disentanglement of natural rubber into the solution. This result could be the effect of the vulcanization of natural rubber molecules in a limited area between the MgCO_3_ particles, which made the crosslink concentrate inside a small area and hindered the dilution from THF, as shown in Figure 3a. In order to understand how MgCO_3_ is incorporated inside a natural rubber matrix, the close-up image of the MgCO_3_ composite is shown in Figure 3b. The MgCO_3_ was squeezed and stacked with natural rubber. It was observed that MgCO_3_ was not found inside the big void in the natural rubber matrix. The density of MgCO_3_ natural rubber with different MgCO_3_ concentrations was reported in Figure 3c. Apparently, with higher MgCO_3_, the density of the composite increased. The morphology of MgCO_3_-filled natural rubber in cross-section is shown in Figure 3d. The pores, which are obviously presented in the matrix of composites, might occur during the curing process in a solid state. Since MgCO_3_ particles were mixed in latex, which contained acetic acid, the mixture could provide CO_2_ gas bubbles during the drying process, where it started to solidify and filled the composite with porosity. These pores became flatter and narrower under the vulcanization process due to shrinkage at elevated temperatures. The dispersion of MgCO_3_ particles in a natural rubber matrix was observed by the energy-dispersive X-ray analyzer (EDX) technique. The mapping of Mg on the surface was illustrated in Figure 3e, which depicted the distribution of Mg over the composite samples. The percentage ratio between Mg and C was 83.61, 87.80, 88.76, 90.38, 90.65, and 92.06 for MgCO_3_ loading of 80, 100, 120, 140, 160, and 180 phr, respectively. These EDX results confirmed the incorporation of MgCO_3_ into the composite matrix because when the loading was higher, the percentage ratio of Mg to C also increased.

**Figure 4:**
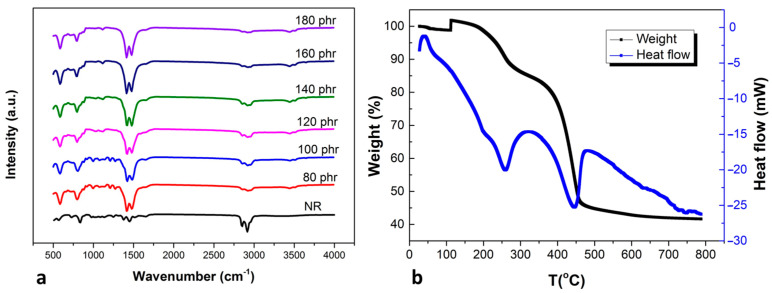
(**a**) FTIR spectra and (**b**) DSC/TGA of 80 phr MgCO_3_ loading.

The bonding properties of MgCO_3_ were investigated by FTIR in Figure 4a, which was recorded in the range of 500–4000 cm^−1^. There were many fingerprint peaks of natural rubber, such as the carbon–carbon double bond bending at 845 cm^−1^, carbon–hydrogen bending at 1452 cm^−1^, and carbon–hydrogen stretching at 2846 and 2915 cm^−1^ [18,19]. Although most of the peaks still persisted after the addition of MgCO_3_, the peak at 797, 1417, and 1480 cm^−1^ were from MgCO_3,_ which did not appear in pure natural rubber and implied the incorporation of MgCO_3_ within the matrix [18,20]. The characteristic peaks of natural rubber at 845 and 2915 cm^−1^ were reduced continuously with a higher amount of MgCO_3_ loading. DSC and TGA in Figure 4b were used to observe the thermal stability of natural rubber composite under nitrogen flow with a heating rate of 10 °C/min. The mass of the composite was beginning to reduce at 100 °C from the evaporation of residual humidity inside the composite. The polyisoprene decomposed at 250 °C, which could be observed from a deep energy well and the mass loss. The mass of the composite drastically decreased again at a temperature above 350 °C, which was the temperature where MgCO_3_ started to oxidize with a broad peak exothermic reaction. The mass of MgCO_3_ particles had been reduced during the oxidization due to the formation of CO and CO_2_. These gases formation was vaporized away, and the remaining substance inside the test crucible was MgO, which had a lower mass.

**Figure 5:**
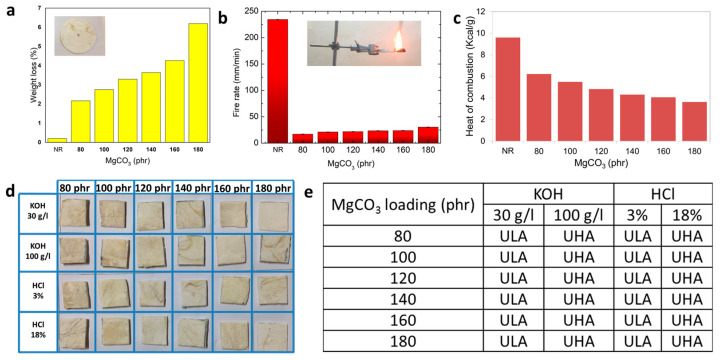
(**a**) Abrasion test; (**b**) flame retardant; (**c**) heat of combustion; and (**d**,**e**) chemical resistance.

Once the composite was formed, the sturdiness of the surface under the physical erosion was studied using the abrasive test, which is shown in Figure 5a. In this test, the weight loss of MgCO_3_/natural rubber composites after testing was measured. The mass loss of pure natural rubber was lowest, and the mass loss of composite was higher when the loading amount of MgCO_3_ was increased. This result implied that a higher amount of inorganic particles on the composite surface might cause a larger amount of particles that could be scratched off. Because the weight loss of the MgCO_3_/NR composite was not much different from the weight loss of the composite that was mixed with other inorganic additives such as Al_2_O_3_ or SiO_2_ (Supplementary Data Figure S1a,b) at the same loading conditions, the cause of weight loss from the abrasion might come from the fabrication process and the properties of natural rubber rather than the types of additive materials.

With a large amount of inorganic additive, the fire retardance property might be significantly different from the usual natural rubber. Fire retardance was the property that was required for modern plastic, especially in building decoration. The characterizations of fire retardance property were performed in Figure 5b, which were examined with the vertical fire rate test. For pure natural rubber, it could easily catch fire, and the burning rate was much higher compared to the composite. When comparing between MgCO_3_ composite, the burning rates were apparently faster with a higher amount of MgCO_3_ loading; however, the fire moving rate was still lower than 40 mm/min, which was under the acceptable value for composite in house decoration. In principle, the flame growing in composite was mainly due to the thermal decomposition of organic natural rubber. In the case of MgCO_3_, the reactive decomposition released CO_2_ with the remnant of MgO particles. Because the reaction was exothermic, the more MgCO_3_ was mixed inside the composite, the faster burning rates were obtained. Consequently, the burning rates of MgCO_3_/NR composite increased with increasing amounts of MgCO_3_ particles. The heat combustion of the composite was conducted by a bomb calorimeter. MgCO_3_/natural rubber composite with the amount of 0.150 g was put in a sealed chamber. After burning, the heat of combustion was recorded in joule per gram and shown in Figure 5c. The energy output of pure natural rubber was very high. When compared between MgCO_3_ composite, despite burning faster in the flame retardance test due to the exothermic reaction of MgCO_3_, the energy output per mass in the bomb calorimeter was reduced when more MgCO_3_ was added into the composite. This less energy output was the result of the mass ratio between natural rubber and MgCO_3_. When burned, it was obvious that inorganic material could provide less energy output compared to organic material.

The abrasion and thermal stability were not the only properties that were a significant concern when used in various conditions. Chemical stability also plays an important role, especially during outdoor application. The composite is supposed to be capable of enduring the chemical attack without changing the surface appearance. The chemical resistance of the MgCO_3_/NR composite is shown in Figure 5d. The chemical resistance of the composite after being immersed in acid and alkali was evaluated by physical appearance. From Figure 5e, the physical appearance was classified into three categories, including no visible effect (LA, HA), visible effects on cut sides, non-cut sides, and on the proper surface (LC, HC). It was noted that “L” and “H” were defined as low concentrations and high concentrations of acid or alkali solution. The results of the test of all MgCO_3_/natural rubber composites were “no visible effect” (LA and HA). Generally, chemical resistance was the ability of composites to withstand chemical attack for a certain time. Because natural rubber was usually produced from the crosslink of multiple isoprene molecules, the crosslinking structure could protect the surface attack from acid or alkali solution even with a very high loading of inorganic particles. 

**Figure 6:**
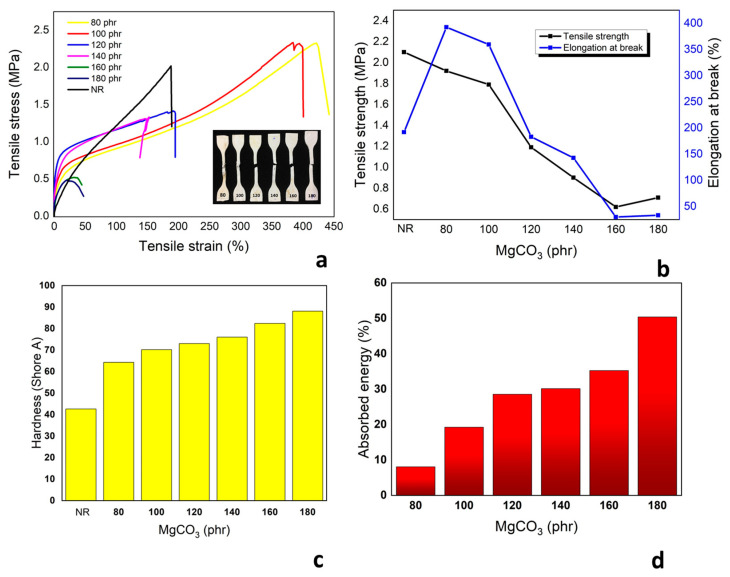
Mechanical properties of MgCO_3_/NR composites; (**a**) stress–strain curve; (**b**) tensile strength and elongation at break; (**c**) hardness; and (**d**) absorbed energy.

A tensile test was used to observe the mechanical property of composites, which is presented in Figure 6a. The yield points of composites were increased until the amount of MgCO_3_ reached 120 phr; however, when more MgCO_3_ was mixed, yield points were dropped. The stress–strain characteristics had completely changed, and composites had lost the elastomeric property. The maximum tensile strengths and maximum elongation of composites continuously decreased when the higher amount of MgCO_3_ particles were loaded, as shown in Figure 6b. Generally, when the additives were filled inside the composites, the strength of the composites was raised due to the particles and polymer chains interaction. On the other hand, in this experiment, the porosities were distributed in the composite matrix, which increased stress concentration and resulted in a reduction of reinforcement efficiency. 

The hardness of composites was scaling up with more amount of MgCO_3,_ as shown in Figure 6c. The maximum hardness of the composite was 88 shore A at 180 phr. It was much higher than the result from the original natural rubber obtained from the same resource, which had hardness around 40 shore A. The result was also higher than the maximum hardness of the composites from Al_2_O_3_ and SiO_2_ (Figure S1c,d), which were produced from the same method. The high hardness was the result of particle and polymer chain interaction and also because MgCO_3_ could be easily blended with latex during the fabrication process. If not because of the high porosity in the matrix, the hardness of the MgCO_3_/NR composite could be much higher. The sponge structure had gaps, and the surface could be deformed, which reduces the obtained hardness. The response of bouncing was observed by dropping a ball with a mass of 50 g in the tube to see the height of the ball after bouncing. The final height and the original height were compared to calculate the absorption energy of the ball in the composite. The result is shown in Figure 6d. The ball could highly bounce up in the composites with a low amount of MgCO_3_ due to the elastomeric property of natural rubber. The bounce heights were continuously reduced with higher energy absorption in the composites, which had a higher amount of MgCO_3_. This was the result of the composite, which lost elastomeric property and was capable of absorbing more energy without bouncing the ball back in the air. This rigid MgCO_3_/NR composite could be used in various applications that need much less elastomeric properties but still prefer natural products.

## 4. Conclusions

We have prepared a non-elastomeric rigid composite from natural rubber by adding a large amount of MgCO_3_. The amount of MgCO_3_ particles in natural rubber latex was added in a wide range from 80 to 180 phr using the solution mixture method. The torque during the MDR test showed a better vulcanization with a higher amount of additive MgCO_3_. MgCO_3_ could be excellently incorporated in the natural rubber matrix; however, the optioned composite had a high porosity due to the CO_2_ formation during the vulcanization process due to the acidic condition of the composite. The lost mass during the abrasion test of composites was higher when the additive amount increased. The flame rate was faster when more MgCO_3_ was due to the exothermic reaction of MgCO_3_, but the rate was still not so high, and energy output was lower when observed from the bomb calorimeter. The composites were sturdy to the acid and alkali solution. The strength and elongation at the break of composites also deteriorated when a very large amount of MgCO_3_ was added, but the hardness was drastically enhanced. At a very high loading of MgCO_3_, the composite lost its elastomeric property and became a rigid material.

## Data Availability

Not applicable.

## Supplementary Materials

The following supporting information can be downloaded at: https://www.mdpi.com/article/10.3390/polym15143078/s1, Figure S1: Abrasion test of (a) Al_2_O_3_/NR composites, (b) SiO_2_/NR composites and Hardness of (c) Al_2_O_3_/NR composites and (d) SiO_2_/NR composites; Table S1: Molecular weight of MgCO_3_/NR composites.
