# Supplementary material for: Natural Rigid and Hard Plastic Fabricated from Elastomeric Degradation of Natural Rubber Composite with Ultra-High Magnesium Carbonate Content
Source: Polymers (Basel). 2023 Jul 18;15(14):3078. doi: 10.3390/polym15143078 (PMC10384260; doi:10.3390/polym15143078)
Supplement: Supplementary file 1 [file polymers-15-03078-s001.zip › polymers-2428504-supplementary.pdf]

Supplementary

Natural Rigid and Hard Plastic Fabricated from Elastomeric Degradation of Natural Rubber Composite with Ultra-High Magnesium Carbonate Content

Abedeen Dasaesamoh, Kittikhun Khotmungkhun and Kittitat Subannajui *

Material Science and Engineering Program, School of Materials Science and Innovation, Faculty of Science, Mahidol University, Bangkok 10400, Thailand; kittikhun.kho@alumni.mahidol.ac.th (K.K.)

* Correspondence: kittitat.sub@mahidol.ac.th

Figure S1. a) Abrassion test of Al_2O_3/NR composites b) Abrasion test of SiO_2/NR composites c) Hardness of Al_2O_3/NR composites d) Hardness of SiO_2/NR composites

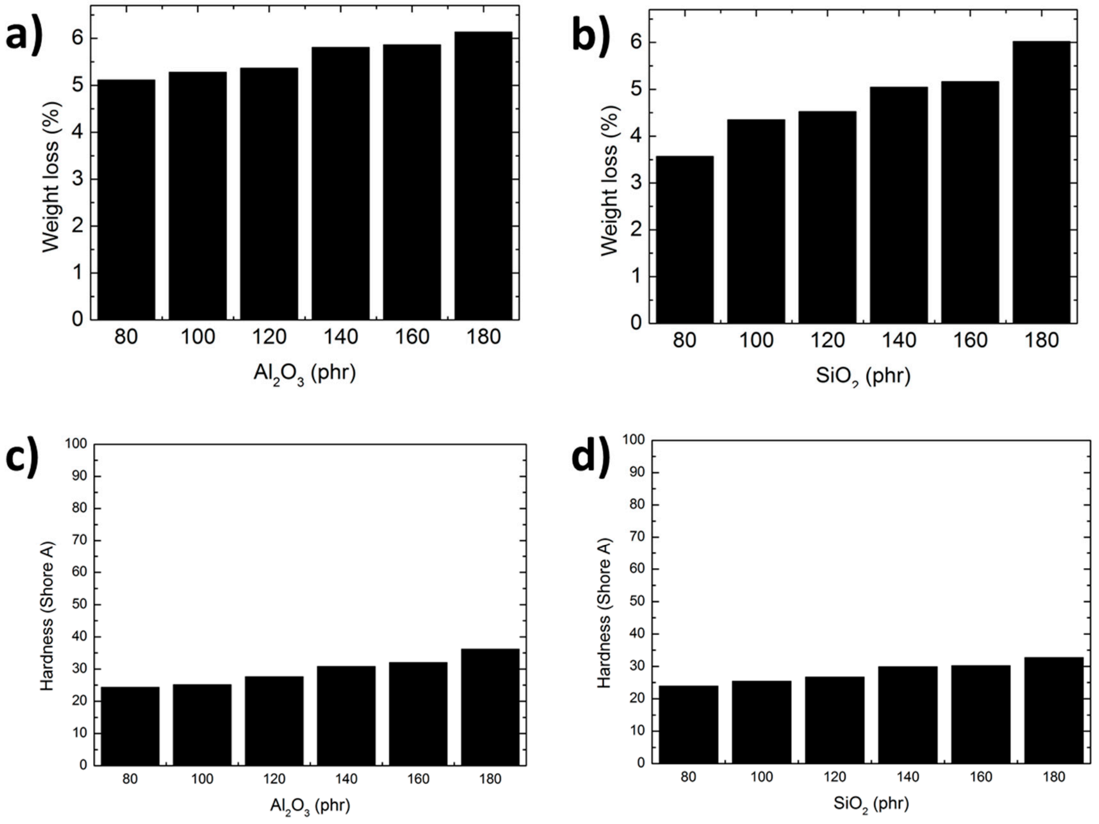

Table S1. The molecular weight of 0 Phr natural rubber measured by GPC-SEC

Molecular weight g/mol	Mp	Mn	Mw	Mz
0 Phr	17387	9043	15198	21690
